# GeniePool 2.0: advancing variant analysis through CHM13-T2T, AlphaMissense, gnomAD V4 integration, and variant co-occurrence queries

**DOI:** 10.1093/database/baae130

**Published:** 2024-12-27

**Authors:** Grisha Weintraub, Noam Hadar, Ehud Gudes, Shlomi Dolev, Ohad S Birk

**Affiliations:** Department of Computer Science, Faculty of Natural Sciences, Ben Gurion University of the Negev, Beer Sheva 84105, Israel; The Morris Kahn Laboratory of Human Genetics at the National Institute of Biotechnology in the Negev and Faculty of Health Sciences, Ben-Gurion University of the Negev, Beer Sheva 84105, Israel; Department of Computer Science, Faculty of Natural Sciences, Ben Gurion University of the Negev, Beer Sheva 84105, Israel; Department of Computer Science, Faculty of Natural Sciences, Ben Gurion University of the Negev, Beer Sheva 84105, Israel; The Morris Kahn Laboratory of Human Genetics at the National Institute of Biotechnology in the Negev and Faculty of Health Sciences, Ben-Gurion University of the Negev, Beer Sheva 84105, Israel

## Abstract

Originally developed to meet the challenges of genomic data deluge, GeniePool emerged as a pioneering platform, enabling efficient storage, accessibility, and analysis of vast genomic datasets, enabled due to its data lake architecture. Building on this foundation, GeniePool 2.0 advances genomic analysis through the integration of cutting-edge variant databases, such as CHM13-T2T, AlphaMissense, and gnomAD V4, coupled with the capability for variant co-occurrence queries. This evolution offers an unprecedented level of granularity and scope in genomic analyses, from enhancing our understanding of variant pathogenicity and phenotypic associations to facilitating research collaborations. The introduction of CHM13-T2T provides a more accurate reference for human genetic variation, AlphaMissense enriches the platform with protein-level impact predictions of missense mutations, and gnomAD V4 offers a comprehensive view of human genetic diversity. Additionally, the innovative feature for variant co-occurrence analysis is pivotal for exploring the combined effects of genetic variations, advancing our comprehension of compound heterozygosity, epistasis, and polygenic risk factors in disease pathogenesis. GeniePool 2.0 is a comprehensive and scalable platform, which aims to enhance genomic data analysis and contribute to genomic research, potentially supporting new discoveries and clinical innovations.

**Database URL**: https://GeniePool.link

## Introduction

The advent of next-generation sequencing (NGS) technologies has revolutionized the field of genomics, unveiling an unprecedented volume of data that holds the key to understanding genetic diseases, evolutionary biology, and personalized medicine. In response to this data deluge, the original GeniePool [1] platform emerged as a pioneering solution, addressing the critical need for efficient storage [2], accessibility [3], and analysis of phenotypic–genotypic information [4, 5]. By harnessing cloud data lake architecture and offering a user-friendly interface, GeniePool made it possible for clinicians and researchers to navigate through vast genomic datasets with ease.

As we transition from GeniePool to GeniePool 2.0, the landscape of genomics has continued to evolve rapidly. The integration of comprehensive variant databases such as CHM13-T2T [6], AlphaMissense [7], and the latest iteration of gnomAD V4, combined with the ability to perform variant co-occurrence queries, represents a significant leap forward. These enhancements not only refine the granularity of genomic analyses but also expand the horizon of research and clinical diagnostics by offering deeper insights into variant pathogenicity, phenotypic associations, and possible research collaboration.

Building on the foundation laid by its predecessor, GeniePool 2.0 is designed to meet the growing demands of the genomics community for more sophisticated analytical tools. The integration of CHM13-T2T provides a more accurate reference for human genetic variation, AlphaMissense enriches the platform with protein-level impact predictions of missense mutations and the incorporation of gnomAD V4 [8, 9] offers a comprehensive view of human genetic diversity. Together, these resources empower users to conduct multifaceted analyses that were previously unattainable, from elucidating complex genotype–phenotype relationships to identifying novel therapeutic targets.

Moreover, GeniePool 2.0 introduces a streamlined feature for variant co-occurrence analysis, enabling researchers to explore the combined effects of genetic variations within individual genomes. This capability is pivotal for understanding compound heterozygosity, epistasis, and polygenic risk factors in disease pathogenesis. Notably, gnomAD offers the same feature with the additional important option to assess whether the two variants are in *cis* or *trans*, but only of hg19 [10] while GeniePool 2.0 also supports hg38 and CHM13-T2T.

GeniePool 2.0 aims to contribute significantly to genomic data analysis by providing a comprehensive, intuitive, and scalable platform tailored to meet the evolving needs of the genomics community. With its enhancements, GeniePool 2.0 seeks to support broader access to genomic research, potentially aiding in discoveries that improve our understanding of genetic mechanisms and their clinical applications.

## Methods

### NGS data preprocessing

NGS samples were preprocessed as previously described [1, 11–13] for hg19 and hg38 data, but for GeniePool 2.0 the CHM13V2.0 [6] reference genome was also used.

### Variant co-occurrence feature development

GeniePool’s data lake contains the VCF files of all preprocessed samples. The feature was implemented using the conjunction between the results of two separate GeniePool API requests (http://api.geniepool.link/rest/index/#reference/#chromosome:#start-#end).

### AlphaMissense integration

AlphaMissense data for hg19 and hg38 were obtained from the links in the AlphaMissense publication [7]. To generate a CHM13V2.0 version, which was not included in the original publication, we utilized Picard toolkit’s LiftoverVcf function (http://broadinstitute.github.io/picard/).

### gnomAD V4 integration

The GeniePool web-application user interface provides a direct link for the gnomAD [14] website for each variant, based on its reference. Even though gnomAD V4 currently does not support CHM13V2.0, we decided to make it available by providing gnomAD links to the corresponding hg38 coordinate page. To obtain all available corresponding gnomAD V4 hg38 coordinates, VCF data for each chromosome was downloaded directly from gnomAD’s website download page (https://gnomad.broadinstitute.org/downloads) and, as described previously with AlphaMissense, we used Picard toolkit’s LiftoverVcf to obtain all hg38 coordinates with gnomAD V4 data that can be remapped to CHM13V2.0. This way, our REST API, when using CHM13V2.0 as reference, returns the hg38 coordinate for each CHM13V2.0 variant when available. The same feature is also available for the older hg19 reference genome, which is still extensively used.

## Results

### Processed data

GeniePool currently houses 60 463 samples for each of the hg19 and hg38 reference genomes, 6601 more samples than in our first publication. Additionally, there are 12 382 CH13V2.0 new samples, accompanied by dbSNP and AlphaMissense variant annotations lifted over from hg38 and corresponding gnomAD V4 links if available.

### Application programming interface

GeniePool’s API requests syntax now supports CHM13V2.0 using the pattern: http://api.geniepool.link/rest/index/chm13v2/chr:start-end. The response format has slightly changed as we added the “AlphaMissense” score for all reference genomes, and in the case of CHM13V2.0, we added the “hg_38_coordinate” field to support GnomAD V4 retrieval. Additional filtration parameters are now available for AlphaMissense scores, variant call quality and coverage. The full details of API changes are available at https://geniepool.link/GeniePool_API_documentation.pdf.

### Web user interface

**Figure 1.**
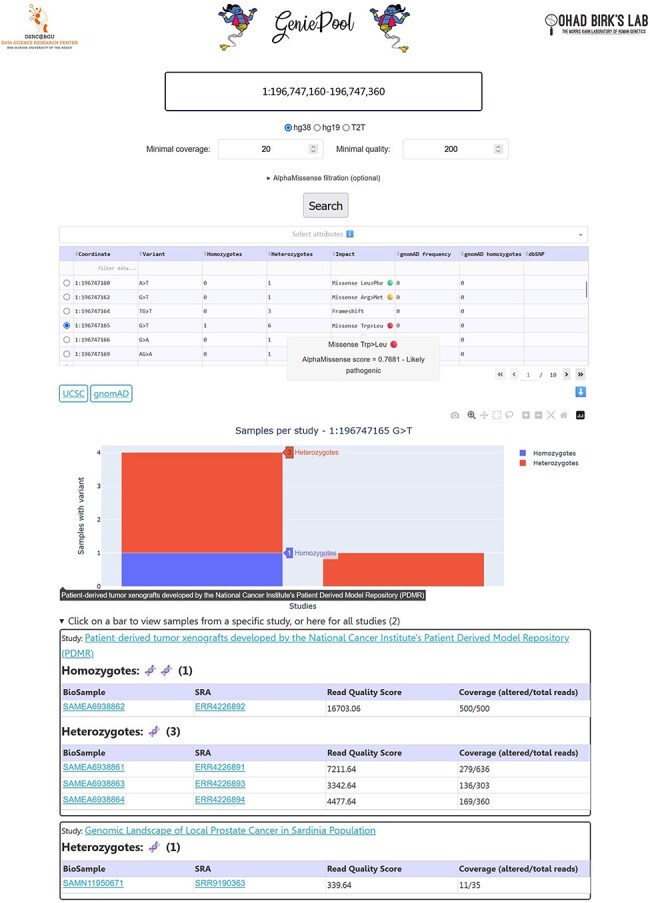
GeniePool 2.0 user interface. Genomic coordinates for either a single variant or co-occurrence of two variants are searched within NGS samples from SRA. Resulting variants can be filtered by sample attributes. Selected variants will provide information about each variant and its study.

GeniePool 2.0 enables the user to choose between two modes of variant queries: “Single variant” (default) or “Two variant co-occurrence.” The latter will output the names of the samples with both variants with links to their corresponding Sequence-Read-Archive (SRA) [4] page followed by a table with additional information regarding the searched variants (Fig. 1).

### Complex queries

Users can perform queries using both the REST-API and the web interface to retrieve variants based on multiple parameters. For genomic locations, users can specify genomic coordinates in a chromosome:position/chromosome:start-end formats, a gene symbol, or dbSNP accessions. Results can also be filtered based on minimum AlphaMissense scores, minimum coverage and quality scores. Using the UI, results can be further filtered based on number of heterozygotes, homozygotes, variant effects, gnomAD homozygote count, and maximal gnomAD allele frequency. Although gnomAD4 primarily supports hg38 and AlphaMissense is not published for CHM13V2.0, we have used Picard’s LiftOver to make these features available for all three reference genomes supported by GeniePool: hg19, hg38, and CHM13V2.0 (T2T).

### Use-case demonstration #1: assessing THBS2 variants as cancer drivers

Key players in the formation of the extra-cellular-matrix (ECM) are matrix-metalloproteinase (MMP) proteins such as MMP2, which breakdown and shape the ECM, primarily in collagen IV-rich tissues [15]. THBS2 binds MMP2 directly and causes its degradation, thus regulating excessive collagen breakdown [16]. Indeed, *THBS2* Knock-out (KO) mice display Ehlers–Danlos syndrome-like features [17]. However, in a recent study [18], mice with a heterozygous *THBS2* missense mutation (and humans with a similar mutation in the orthologous gene) were found to display the very same phenotypes as in the previously reported homozygous KO mice. As the heterozygous missense mutation caused a phenotype similar to that of the homozygous KO mice, along with the fact that THBS2 is a homo-trimer, we hypothesized that one or two mutated THBS2 proteins can bind wild-type ones and cause a dominant-negative effect.

To further inspect our hypothesis, we aimed to find other examples with various *THBS2* missense variants that are expected to disrupt the protein’s structure drastically, and then learn about their properties. To perform such analysis, we used GeniePool *2.0* to identify coding variants within *THBS2* and keep only missense variants with reasonable sequencing quality (score ≥100) and coverage (≥20 read sequences), as well as the highest AlphaMissense score to drastically alter the protein formation (score ≥ 0.99). For the 104 variants identified, we then inspected the number of heterozygotes and homozygotes in gnomAD for each variant. Notably, not a single homozygote could be found; moreover, there were only 8 different heterozygous *THBS2* in gnomAD, with one variant having 10 heterozygotes, two variants having 3 heterozygotes, one variant with 2 heterozygotes and four variants with a single heterozygote each. Using GeniePool 2.0, we looked up the details for each of the samples’ BioProject and BioSample data and discovered that 93 of them were discovered in whole-exome-sequencing of tumor samples, 12 in nontumor samples (3 variants with both tumor and nontumor samples) and 2 variants in samples whose descriptions were insufficient to decide with certainty whether they came from a tumor or a nontumor tissue (Supplementary Table S1).

### Use-case demonstration #2: in silico assessment of additional gene variation effects

*O*-glycans are sugar complexes that play an important role in autoimmunity regulation, among other functions. *O*-glycans are generated from different saccharides, known as “Cores.” The Core-1 residue, when exposed, is known as the “Tn-antigen” while the exposed Core-2 residue is called “T-antigen.” Normally, the two antigens are covered by other sugar residues, mainly sialic acid. Exposed T- and Tn-antigens can cause autoimmune conditions [19, 20].

The gene *C1GALT1C1* encodes a chaperone which is crucial for the formation of the T-antigen on top of the Tn-antigen. One might expect a *C1GALT1C1* mutation to cause Tn-antigen but not T-antigen exposure, as it will not allow the formation of the Core-2 on top of Core-1. Recently, two different publications described pathogenic *C1GALT1C1* mutations, each in a different amino acid, resulting in different glycosylation patterns; The first mutation [19] (p.T89I) was shown to cause T-antigen but not Tn-antigen exposure, while the other [20] (p.Ala20Asp) causes exposure of both, but mainly of Tn-antigen.

We utilized GeniePool 2.0 to gain a broader view on samples with *C1GALT1C1* missense variants using the same methodology previously described with *THBS2*, except for allowing AlphaMissense to have the minimal value of “likely pathogenic” (≥ 0.564). The analysis yielded 87 variants in 1282 samples from 244 studies (Supplementary Table S2). Intriguingly, six different variants appeared in the same study (NCBI accession SRP186739) entitled “Inferring evolution and progression of small-cell lung cancer by single-cell Sequencing of circulating tumor cells” [21] that might suggest a connection between *C1GALT1C1* and small-cell lung cancer, in accordance with previous studies [22–24].

## Discussion

GeniePool 2.0 embodies the forefront of genomic data analysis by leveraging the latest advancements in genomic databases and bioinformatic tools. With the feature for variant co-occurrence queries, its integration of CHM13-T2T [6], AlphaMissense [7], and gnomAD V4 [8, 9], GeniePool 2.0 significantly enhances the precision and scope of genomic analyses. This leap forward underscores the platform’s commitment to keeping pace with the rapid evolution of genomics, providing a critical resource for researchers and clinicians alike. We demonstrated its effectiveness in further studies of variants and genes in ways far beyond the mere assessment of their population frequency, as usually done by traditional genomic databases, and expect users to use GeniePool in versatile and creative ways to explore genetics and medicine.

The early adoption and integration of the CHM13-T2T reference genome is particularly noteworthy. This initiative places GeniePool 2.0 at the forefront of the field, as the T2T project represents a complete human genome sequence, filling in gaps left by previous reference genomes. GeniePool is also easily integrated into other platforms, and was already adopted as a feature for further variant investigation in VARista [25] (https://VARista.link), a variant analysis web-application for VCF files. This strategic move anticipates future trends in genomics, ensuring that GeniePool remains a valuable and up-to-date resource for its users.

Looking forward, the potential of integrating large language models (LLMs) such as ChatGPT [26] for performing complex queries that span both genotypes and phenotypes data stored in our data lake is both exciting and challenging [27]. Despite the transformative impact of LLMs on various fields, their application in genomics is still not mature enough. Our current experiences with LLMs such as ChatGPT4 [26] and Llama 2 [28] have revealed limitations, including inconsistent responses and inaccuracies. However, the evolving capabilities of LLMs promise to revolutionize the way genomic data are queried and analyzed. This could enable sophisticated, intuitive querying mechanisms for researchers seeking to unravel the complex interplay between genotypes and phenotypes, and we expect genomic data bases built upon data lake architecture to stand at the forefront of this revolution.

In conclusion, GeniePool 2.0 aims to contribute to innovation in genomics by incorporating the latest genomic references and integrating emerging technologies such as LLMs. While striving to keep pace with advancements in the field, GeniePool 2.0 seeks to facilitate the broader application of genomics in research and clinical settings. Looking forward, the ongoing development of GeniePool and similar platforms could play a key role in harnessing the full potential of genomics to enhance our understanding of genetic foundations.

## Supplementary Material

baae130_Supp

## Data Availability

GeniePool web UI is available at: https://GeniePool.link. The GeniePool source code, including a Python 3 script that automates the generation of tables similar to Supplementary Tables S1 and S2, is available in the GitHub repository (https://github.com/geniepool). REST API is available using: http://api.geniepool.link/rest/index/$reference/$coordinates (with chm13v2/hg38/hg19 for reference and chr:start-end for coordinates, e.g. http://api.geniepool.link/rest/index/chm13v2/1:12345789-123456798). Additional filtration parameters are now available for AlphaMissense scores, variant call quality and coverage. API documentation is available at: https://geniepool.link/GeniePool_API_documentation.pdf.
